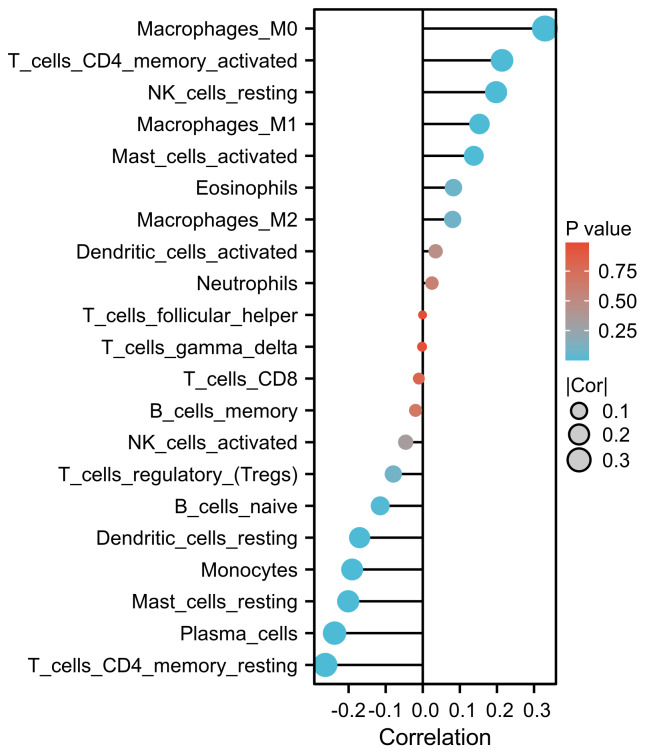# The prognostic value of bioinformatics analysis of ECM receptor signaling pathways and LAMB1 identification as a promising prognostic biomarker of lung adenocarcinoma

**DOI:** 10.1097/MD.0000000000039854

**Published:** 2024-09-20

**Authors:** Tingjun Liu, Jing Liu, Quangang Chen, Lianlian Wu, Lingzhi Zhang, Dandan Qiao, Zhutao Huang, Tianyuan Lu, Ankang Hu, Jie Wang

**Affiliations:** a Center of Animal Laboratory, Xuzhou Medical University, Xuzhou, Jiangsu, China; b Department of Respiratory Medicine, Xuzhou Central Hospital, Xuzhou, Jiangsu, China.

**Keywords:** extracellular matrix receptor interaction, lung adenocarcinoma, prognostic model, tumor immune microenvironment

## Abstract

The extracellular matrix (ECM) is a complex and dynamic network of cross-linked proteins and a fundamental building block in multicellular organisms. Our study investigates the impact of genes related to the ECM receptor interaction pathway on immune-targeted therapy and lung adenocarcinoma (LUAD) prognosis. This study obtained LUAD chip data (GSE68465, GSE31210, and GSE116959) from NCBI GEO. Moreover, the gene data associated with the ECM receptor interaction pathway was downloaded from the Molecular Signature Database. Differentially expressed genes were identified using GEO2R, followed by analyzing their correlation with immune cell infiltration. Univariate Cox regression analysis screened out ECM-related genes significantly related to the survival prognosis of LUAD patients. Additionally, Lasso regression and multivariate Cox regression analysis helped construct a prognostic model. Patients were stratified by risk score and survival analyses. The prognostic models were evaluated using receiver operating characteristic curves, and risk scores and prognosis associations were analyzed using univariate and multivariate Cox regression analyses. A core gene was selected for gene set enrichment analysis and CIBERSORT analysis to determine its function and tumor-infiltrating immune cell proportion, respectively. The results revealed that the most abundant pathways among differentially expressed genes in LUAD primarily involved the cell cycle, ECM receptor interaction, protein digestion and absorption, p53 signaling pathway, complement and coagulation cascade, and tyrosine metabolism. Two ECM-associated subtypes were identified by consensus clustering. Besides, an ECM-related prognostic model was validated to predict LUAD survival, and it was associated with the tumor immune microenvironment. Additional cross-analysis screened laminin subunit beta 1 (LAMB1) for further research. The survival time of LUAD patients with elevated LAMB1 expression was longer than those with low LAMB1 expression. Gene set enrichment analysis and CIBERSORT analyses revealed that LAMB1 expression correlated with tumor immune microenvironment. In conclusion, a prognostic model of LUAD patients depending on the ECM receptor interaction pathway was constructed. Screening out LAMB1 can become a prognostic risk factor for LUAD patients or a potential target during LUAD treatment.

## 1. Introduction

Lung cancer has the highest morbidity and mortality globally, with about 1.6 million people deaths every year.^[1]^ Non-small cell lung cancer accounts for more than 80% of lung cancers, with lung adenocarcinoma (LUAD) being the most diagnosed subtype.^[2]^ Although comprehensive chemotherapy, radiotherapy, and molecular-targeted therapy benefit patients, the 5-year survival rate is only 15%.^[3]^ Therefore, there is an urgent need to explore novel biomarkers to determine the prognosis of LUAD patients and prolong their survival.

The extracellular matrix (ECM) is a complex and dynamic network of cross-linked proteins and a fundamental building block in multicellular organisms.^[4]^ The ECM coordinates cellular processes, including adhesion, migration, proliferation, survival, and differentiation. ECM remodeling is associated with cross-linking of tumor, stromal, and immune cells between primary and secondary sites.^[5,6]^ Conversely, destroying ECM tissue and changing basic components can regulate cancer initiation and metastasis by enhancing tumor-associated angiogenesis and inflammation.^[7]^ The extracellular ECM localization makes it ideal for imaging and targeting because probes can access the ECM without crossing the cell membrane. ECM-targeting nanobodies have been successfully utilized for PET/CT imaging of primary tumors and metastases within several cancer models.^[8]^ The ECM receptor interaction signaling pathway is a critical signal transduction pathway in cells. This plays a vital role in tumor shedding, adhesion, degradation, movement, and proliferation.^[9]^ Studies have indicated that the ECM and glioblastoma microenvironment interaction is critical for abnormal angiogenesis and diffuse glioblastoma infiltration.^[10]^ However, whether these ECM receptor signaling pathway-related genes are associated with LUAD patient prognosis remains unknown.

This study used bioinformatics methods to analyze the correlation between some ECM receptor signaling pathway-related genes and LUAD prognosis, immune cell infiltration, etc. Moreover, a risk prediction model was developed to explore the ECM receptor signaling pathway significance in LUAD.

## 2. Materials and methods

### 2.1. Cell culture

A549, H1299, H1975, PC9, HCC78, and BEAS-2B cells were cultured in DMEM medium supplemented with 10% fetal bovine serum, 1% penicillin, and streptomycin at 37 °C with 5% CO_2_. The cells were divided into a control group and an experimental group. Experiments were conducted when the cells reached the logarithmic growth phase.

### 2.2. Western blotting

A549, H1299, H1975, PC9, HCC78, and BEAS-2B cells were digested with trypsin, washed with PBS, and lysed with RIPA lysis buffer (lysed on ice for 30 minutes) (Beyotime). The BCA method was used to determine protein concentration. Equal amounts of protein were loaded onto the gel for separation and transferred to a Hybond NC membrane (VICMED). The membrane was blocked with 5% skim milk for 1 hour at room temperature, followed by overnight incubation with primary antibodies of anti-laminin subunit beta 1 (anti-LAMB1) antibody (ab109293, 1:1000, Abcam) at 4 °C. The next day, the membrane was washed and peroxidized with appropriate horseradish at room temperature. Subsequently, the blot was incubated with HRP-conjugated secondary antibody (RGAR001, 1:3000, Proteintech) for 1 hour using a chemiluminescent detection system ((Bio-Rad, USA).

### 2.3. Data collection and processing

**Figure 1.**
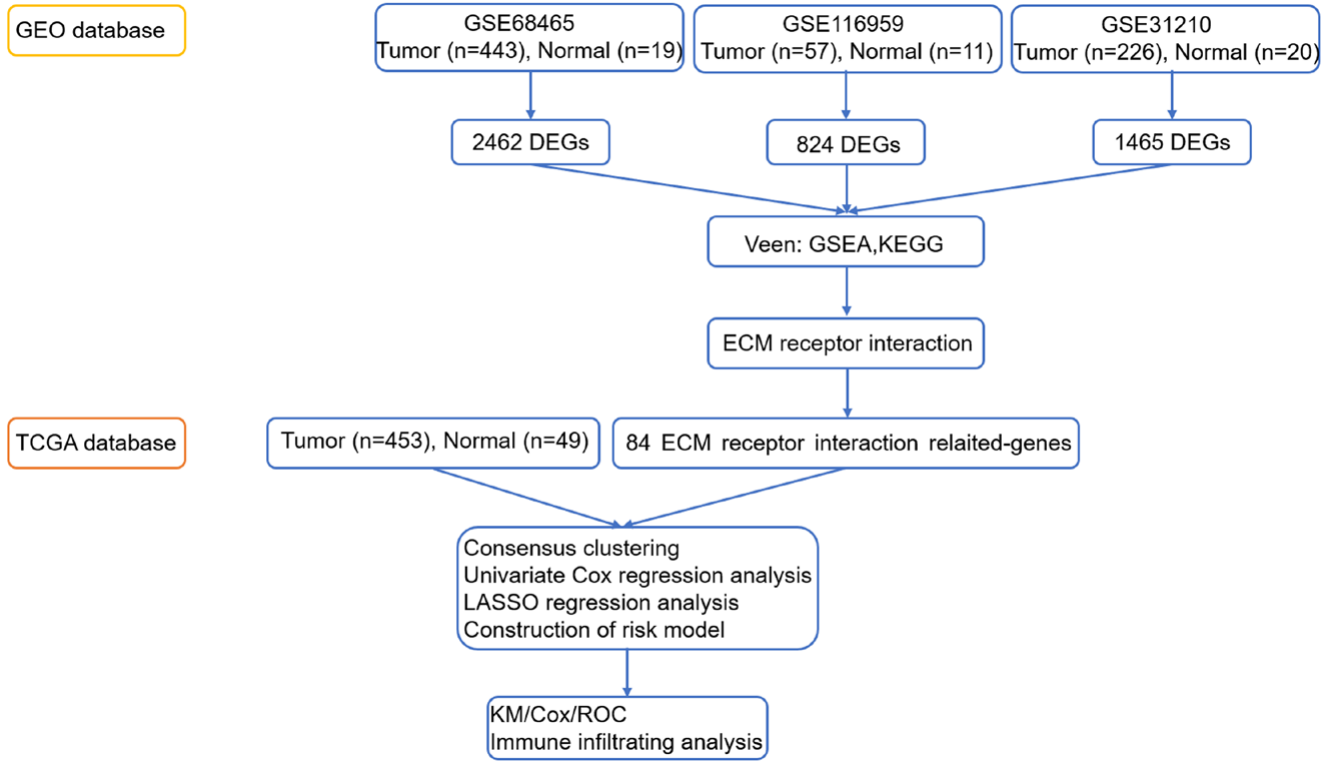
Patient flow diagram, clinical significance, and disclosure statement for this manuscript.

We sourced 3 independent datasets (GSE684645, GSE31210, and GSE116959) from the National Center for Biotechnology Information database NCBI (https://www.ncbi.nlm.nih.gov/). The TPM of LUAD and normal tissue were downloaded from The Cancer Genome Atlas (TCGA) database website. Later the raw data was extracted into a matrix file using the Perl software, and the gene IDs in the raw data were converted to gene names. TCGA and Gene Expression Omnibus (GEO) data were integrated into the dataset with the “sva” package. The 84 ECM-related genes were retrieved from the Molecular Signature Database (MsigDB) (https://www.gsea-msigdb.org/gsea/msigdb/index.jsp). The whole process of data analysis is shown in Figure 1.

### 2.4. Cluster consistency analysis

Consensus clustering differentiates common cancer-type samples into subtypes based on different omics datasets to discover or compare new disease subtypes. The process was repeated 1000 times to determine the optimal cluster number in the k = 2 to 10 range and to ensure the outcome stability.

### 2.5. Identification and functional enrichment analysis of differentially expressed genes

The Limma R software package (version: 3.40.2) helped evaluate differential mRNA expressions. The screening criteria for differential mRNA expressions were adjusted at *P* < .05 and |fold change| > 2. Gene Ontology (GO) and Kyoto Encyclopedia of Genes and Genomes (KEGG) facilitated the analysis and comparison of differential signaling pathways and biological effects between LUAD cancer and normal samples. The “clusterProfiler” package in R software helped evaluate the GO and KEGG pathways. The enriched items with *P* < .05 and Q < 0.05 were statistically significant, which could be visualized using bubble and circle plots.

### 2.6. Gene set enrichment analysis (GSEA)

GSEA was performed using the GSEAv4.1.0 software to identify biological pathways related to the high and low expression of LAMB1 in LUAD cancer and normal samples. *P* < .05 depicts statistical significance.

### 2.7. Construction of prognostic model and evaluation of prognostic performance

Univariate Cox regression analysis helped determine the correlation between ECM-related genes and LUAD prognosis while screening out the genes associated with survival. Using the Lasso–Cox regression model for 10-fold cross-validation, we obtained ECM-related genes significantly correlated with overall survival (OS), and a prognostic model was developed. The risk score is calculated as (regression coefficient of ECM-related gene expression) +... + (regression coefficient of ECM-related gene expression). Based on the median division of the risk value of the prognostic model, the LUAD samples were categorized into high-risk and low-risk groups, followed by Kaplan–Meier survival analysis. A Receiver Operating Curve helped evaluate the predictive power. *P* < .05 demonstrates statistical significance.

### 2.8. Immune microenvironment analysis

The ESTIMATE algorithm helped determine the proportion of immune and stromal cells within the tumor microenvironment (TME) of tumor samples. Higher scores denote a more significant proportion of immune or stromal cells. We used the single-sample gene enrichment analysis (ssGSEA) method to determine the infiltration degree of 22 types of immune cells in LUAD tumor tissue. Moreover, the Spearman correlation coefficient was calculated between various immune cells and LAMB1 gene expression while establishing the relationship between the LAMB1 gene and immune cell infiltration. The “violet,” “ggplot2,” and “ggpubr” R packages helped make scatterplots, boxplots, and violin plots to investigate the Spearman correlation coefficients between gene expression and multiple immune checkpoint molecules and T cell effectors.

### 2.9. Statistical analysis

We performed statistical analyses using R (version 4.1.3) and GraphPad Prism 8. Logistic regression analysis was conducted in SPSS (version 26.0). Two groups were analyzed using the Student *t* test, and more than 2 groups were analyzed with a one-way analysis of variance. *P* < .05 indicates statistical significance, and all the analyses were performed according to the two-sided tests (“*”: *P* < .05. “**”: *P* < .01. “***”: *P* < .001. “*****”: *P* < .0001. “-”: not significant).

## 3. Results

### 3.1. Differentially expressed genes (DEGs) and functional enrichment analysis in LUAD

**Figure 2.**
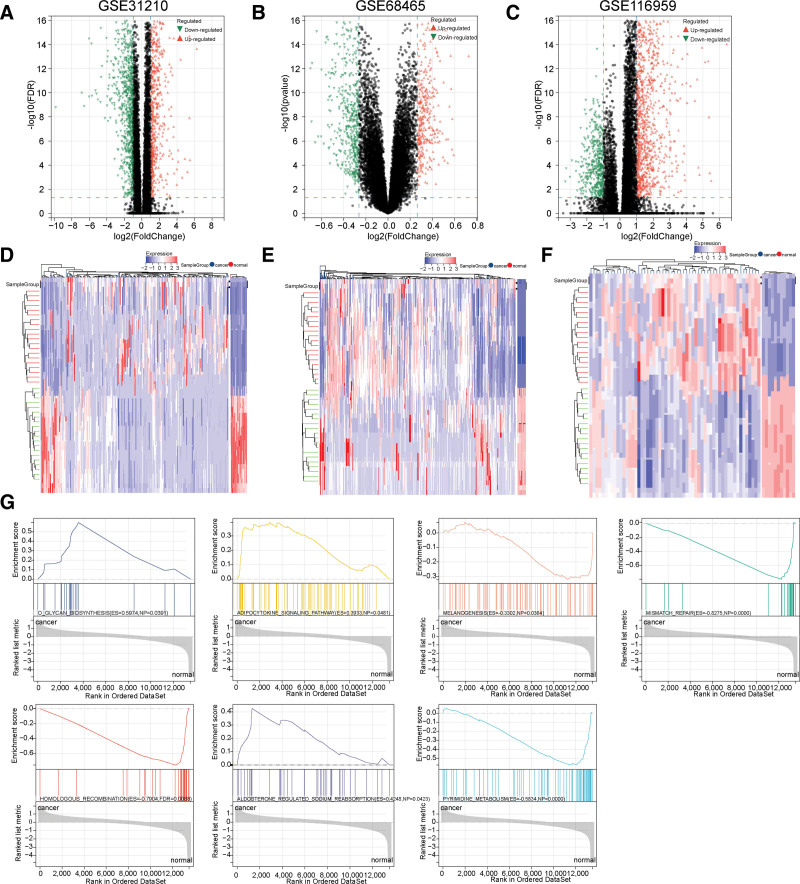
The differentially expressed genes (DEGs) and GSEA pathway analysis in LUAD. (A–C) The Volcano plot describes the expression profiles of DEGs in normal and LUAD samples from the GSE31210, GSE68465, and GSE116959 cohorts. (D–F) The heatmap illustrates the expression profiles of DEGs. (G) GSEA profiles depict the 7 significant GSEA sets associated with DEGs.

We obtained 3 LUAD-related GEO datasets, GSE31210, GSE68465, and GSE116959, from the NCBI GEO database for subsequent analysis. The difference analysis revealed 1465 DEGs in the GSE31210 dataset compared with normal samples. Among them, 1086 genes were up-regulated, and 379 were down-regulated. There were 2462 DEGs in the GSE68465 dataset, with 1657 up-regulated and 805 down-regulated genes. There were 824 DEGs in the GSE116959 dataset, with 492 up-regulated and 332 down-regulated genes, visualized as volcano and heat maps (Fig. 2A–F) (Tables S1–S3, Supplemental Digital Content, http://links.lww.com/MD/N647). GSEA analysis demonstrated that DEGs from these 3 datasets were primarily involved in homologous recombination, pyrimidine metabolism, mismatch repair, O-glycan biosynthesis, melanin formation, adipocytokine signaling pathway, and aldosterone-mediated iodine uptake (Fig. 2G) (Table S4, Supplemental Digital Content, http://links.lww.com/MD/N647). KEGG pathway analysis helped process the DEGs for functional enrichment analysis. We observed that the pathways associated with DEGs primarily involved cell cycle, ECM receptor interaction, protein digestion and absorption, p53 signaling pathway, complement and coagulation level linkage, tyrosine metabolism, etc. (Table S5, Supplemental Digital Content, http://links.lww.com/MD/N647).

### 3.2. Molecular typing of ECM-related genes and analysis of differentially expressed genes and signaling pathways of various subtypes

**Figure 3.**
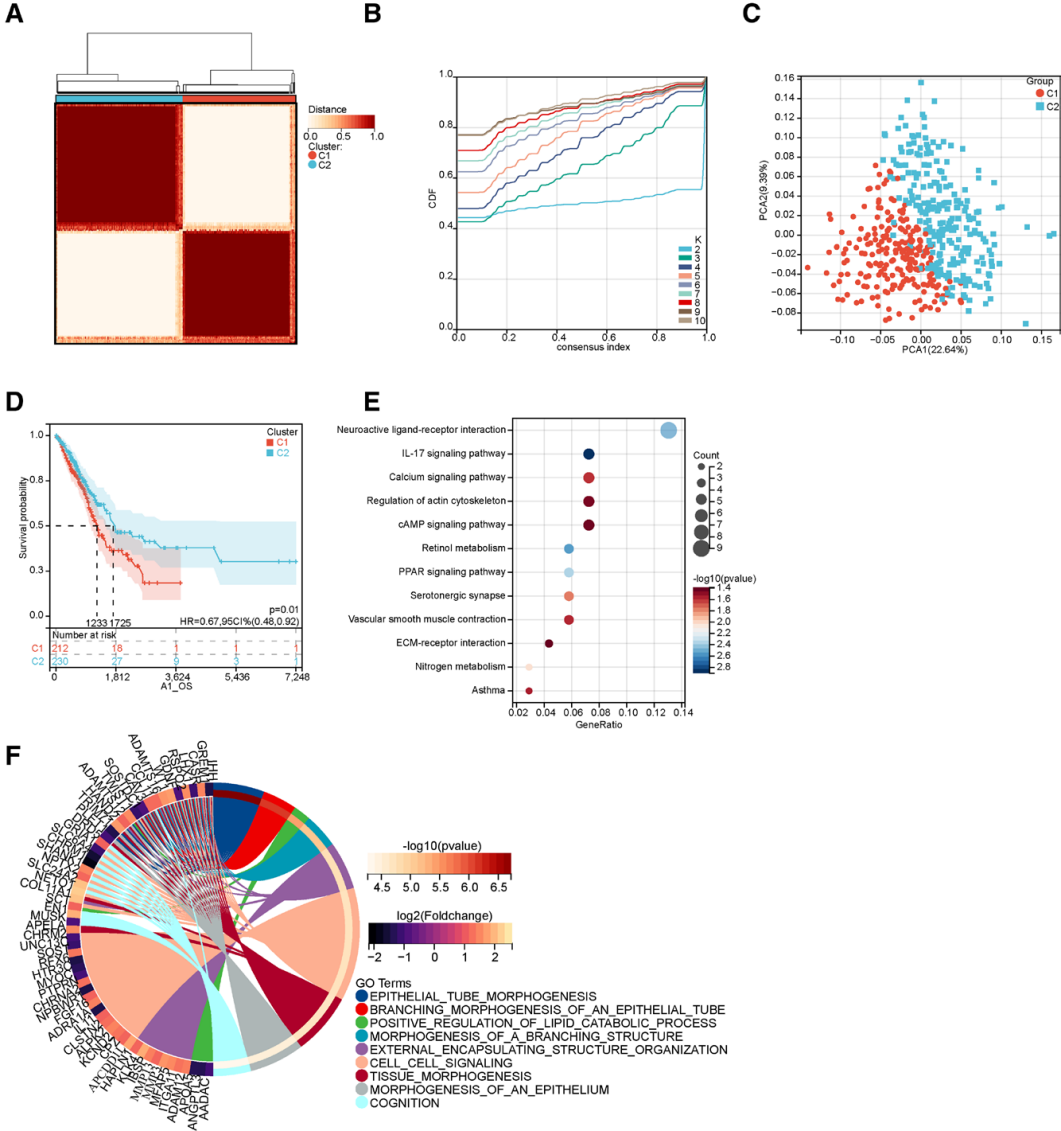
The identification of ECM receptor-interacting pathway subtypes and their signaling pathways using cluster analysis. (A) The heatmap describes the consensus clustering solution (k = 2) for 84 genes in 453 LUAD samples. (B) The delta area curve of consensus clustering indicates the relative change in the area under the cumulative distribution function (CDF) curve for k = 2–10. (C) The principal component analysis (PCA) of the clusters. (D) The Kaplan–Meier OS curves for the 2 subtypes. (E) The dot plots show 552 DEGs enriched by KEGG. The point size represents the number of genes, and the point color characterizes – log10 (p.adjust-value). (F) The circular plot of 552 DEGs enriched by GO.

We obtained 84 genes associated with the ECM receptor interaction pathways from the MSigDB. According to the expression profiles of the ECM-related genes, cluster analysis was performed on 453 LUAD samples within the TCGA database. After adjusting the clustering value (k) from 2 to 10, the correlation in the LUAD sample was the highest when k = 2, with relatively stable clustering results (Fig. 3A–C). Cluster 1 revealed increased expression of ECM-related genes, while Cluster 2 had low expression of ECM-related genes. The survival time of Cluster 1 was significantly shorter than Cluster 2 (*P* < .05) (Fig. 3D). Next, the DEGs and the signaling pathways they participated in were analyzed in these 2 subtypes. A total of 552 differential genes were obtained, including 384 down-regulated and 168 up-regulated genes (Table S6, Supplemental Digital Content, http://links.lww.com/MD/N647). GO functional enrichment and KEGG pathway analyses primarily enriched these genes in the IL-17, calcium, cAMP, and PPAR signaling pathways, retinol and nitrogen metabolism, serotonergic synapse, and other pathways (Fig. 3E and F).

### 3.3. Analysis of different subtypes of the immune microenvironment

**Figure 4.**
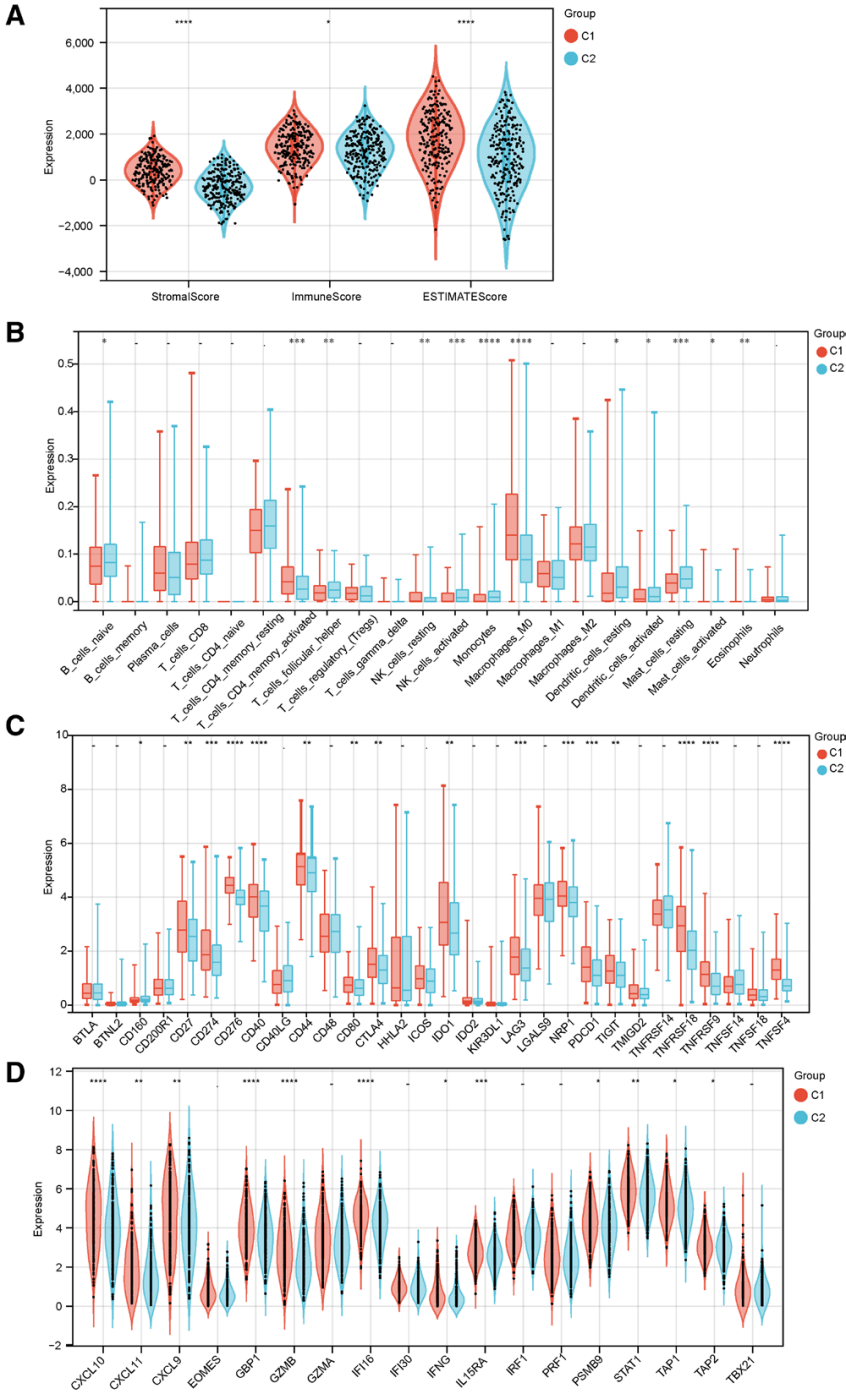
The analysis of the immune microenvironment of various subtypes. (A) The Violin plots indicate the stromal, immune, and ESTIMATE scores. (B) The relative proportion of immune infiltration within different subtypes. (C and D) The Box and Violin plots represent the differential expression of multiple immune checkpoints and T cell effectors in the 2 subtypes. “*”: *P* < .05. “**”: *P* < .01. “***”: *P* < .001. “*****”: *P* < .0001. “–”: not significant.

More evidence depicts that ECM receptor interaction-related pathways are essential in antitumor immunity. Our study deciphered the composition of the TME into 2 subtypes. The results indicated that the ECM-high subtype (Cluster 1) had a higher immune score than the ECM-low subtype (Cluster 2) (Fig. 4A). We employed the CIBERSORT algorithm and the LM22 signature matrix to assess differences in immune cell infiltration between the 2 subtypes. As shown in Figure 4B, the low ECM subtype patients had significantly elevated proportions of naive B cells, follicular T helper cells, activated NK cells, monocytes, resting dendritic cells, and resting mast cells. Moreover, most human T cell effectors and immune checkpoints were up-regulated in the ECM-high subtype. In contrast, the opposite trend was observed within the ECM-low subtype (Fig. 4C and D). Therefore, the ECM-high subtype is related to the immune hot phenotype, whereas the ECM-low subtype is correlated with the cold one.

### 3.4. Construction and evaluation of a prognostic model of ECM receptor interaction pathway

**Figure 5.**
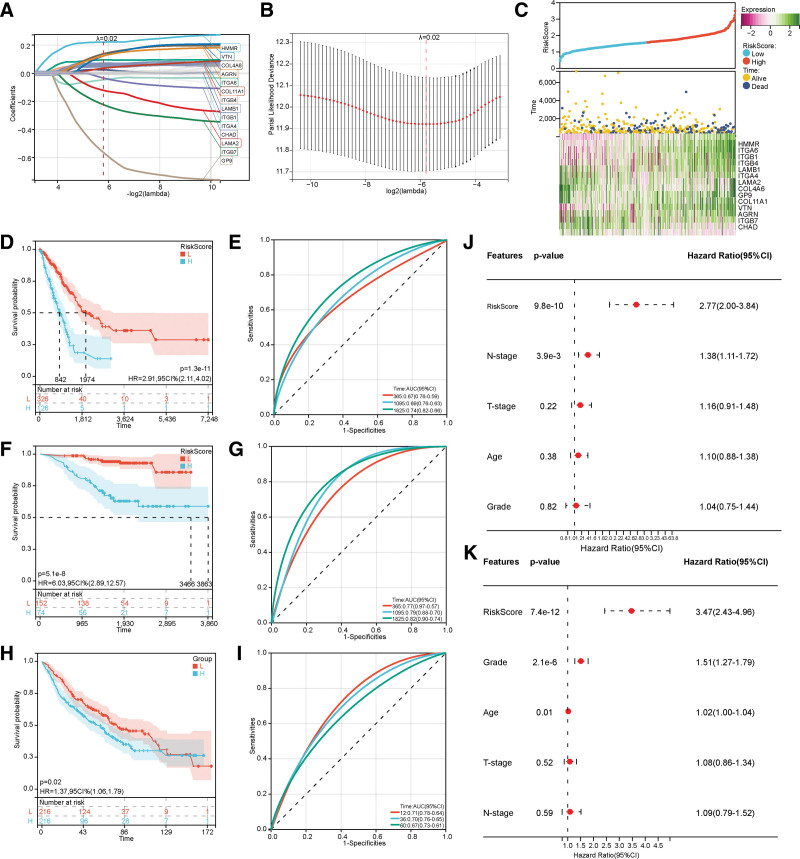
The construction and verification of the ECM risk model. (A and B) Lasso–Cox analysis identified 14 genes correlated with OS in the TCGA dataset. (C) Risk scores distribution, survival status of each patient, and heatmaps of prognostic 14-gene signature in the TCGA database. (D, F, H) The Kaplan–Meier analyses demonstrate the prognostic significance of the risk model. (E, G, I) The time-dependent Receiver Operating Curve analyses for the risk model in the TCGA, GSE31210, and GSE68465 cohort, respectively. (J and K) Univariate and multivariate Cox analyses assess the independent prognostic value of ECM risk signature among LUAD patients.

After univariate Cox regression analysis, 25 survival-related genes were significantly associated with patient OS (Table S7, Supplemental Digital Content, http://links.lww.com/MD/N647). Then, LASSO regression analysis helped reduce the dimensionality of these 25 genes. Finally, 14 genes were screened out, all included in the risk model (Fig. 5A and B). In addition, the relationship between survival status and risk score was also investigated. The study results indicated that the survival status was significantly higher in the low-risk group than in the high-risk one (Fig. 5C). The predictive significance of the risk status in LUAD was determined using KM analysis, with high-risk scores corresponding to poorer OS (Fig. 5D). The areas under the Receiver Operating Curve for overall survival at 1, 3, and 5 years were 0.67, 0.69, and 0.74, respectively (Fig. 5E). This was verified in the GEO dataset GSE31210 (Fig. 5F and G) and GSE68465 (Fig. 5H and I).

Univariate and multivariate Cox analyses depicted that the risk score (*P* < .001) was an independent prognostic factor (Fig. 5J and K). We investigated the association between ECM risk score and TME due to the critical role of ECM in the antitumor immune response. The patients with higher risk scores were negatively associated with monocytes, plasma cells, resting dendritic cells, resting mast cells, and resting memory CD4 T cells. They were also associated with activated dendritic, mast, and memory CD4 T cells. Cells, M0 macrophages, M1 macrophages, and resting NK cells were positively associated (Figure S1, Supplemental Digital Content, http://links.lww.com/MD/N646).

### 3.5. Differential expression, survival, and related pathway analysis of LAMB1 in LUAD

**Figure 6.**
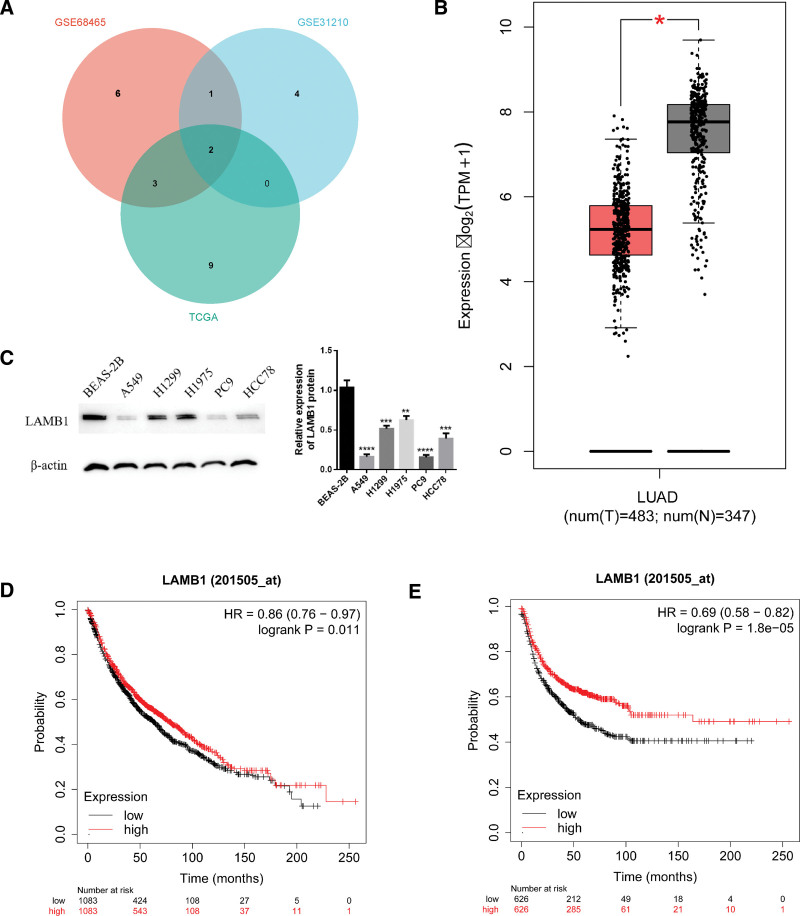
LAMB1 expression and GSEA pathway analysis in LUAD. (A) A Venn diagram of risk-related genes in the 3 cohorts. (B) Differential LAMB1 expression levels within the tumor and normal LUAD tissues based on the TCGA database. “*”: *P* < .05. (C) The expression of LAMB1 in multiple LUAD cell lines and BEAS-2B cells. “*”: *P* < .05. “**”: *P* < .01. “***”: *P* < .001. (D and E) The prognosis of LAMB1 expression for OS and progression-free survival (PFS) among LUAD patients.

We performed LASSO regression analysis, and the GSE68465 and GSE31210 datasets were processed. The results indicated that 12 and 7 genes were included in the risk models in the 2 datasets, respectively (Table S8, Supplemental Digital Content, http://links.lww.com/MD/N647). By displaying the Venn diagram, we identified 2 risk-related genes within the 3 cohorts, viz., LAMB1 and integrin subunit beta 1 (ITGB1) (Fig. 6A). Studies have established that ITGB1 is a prognostic marker for LUAD. Therefore, LAMB1 was selected as the object of further research. The LAMB1 gene showed a down-regulated expression pattern in LUAD by analyzing its mRNA expression level from LUAD samples in the TCGA database (Fig. 6B). The expression of the LAMB1 gene was analyzed in 5 human lung adenocarcinoma cell lines using Western blotting. The WB results indicate that the expression of the LAMB1 protein is down-regulated in human lung adenocarcinoma cell lines A549, H1299, H1975, HCC78, and PC9 compared to normal bronchial epithelial cells (BEAS-2B). (Fig. 6C). In addition, Kaplan–Meier survival analysis revealed that the OS and progression-free survival within the LAMB1 gene low-expression group of LUAD patients were significantly lower than those in the LAMB1 gene high-expression group (*P* < .05) (Fig. 6D and E).

### 3.6. The relationship between LAMB1 expression and the immune microenvironment

**Figure 7.**
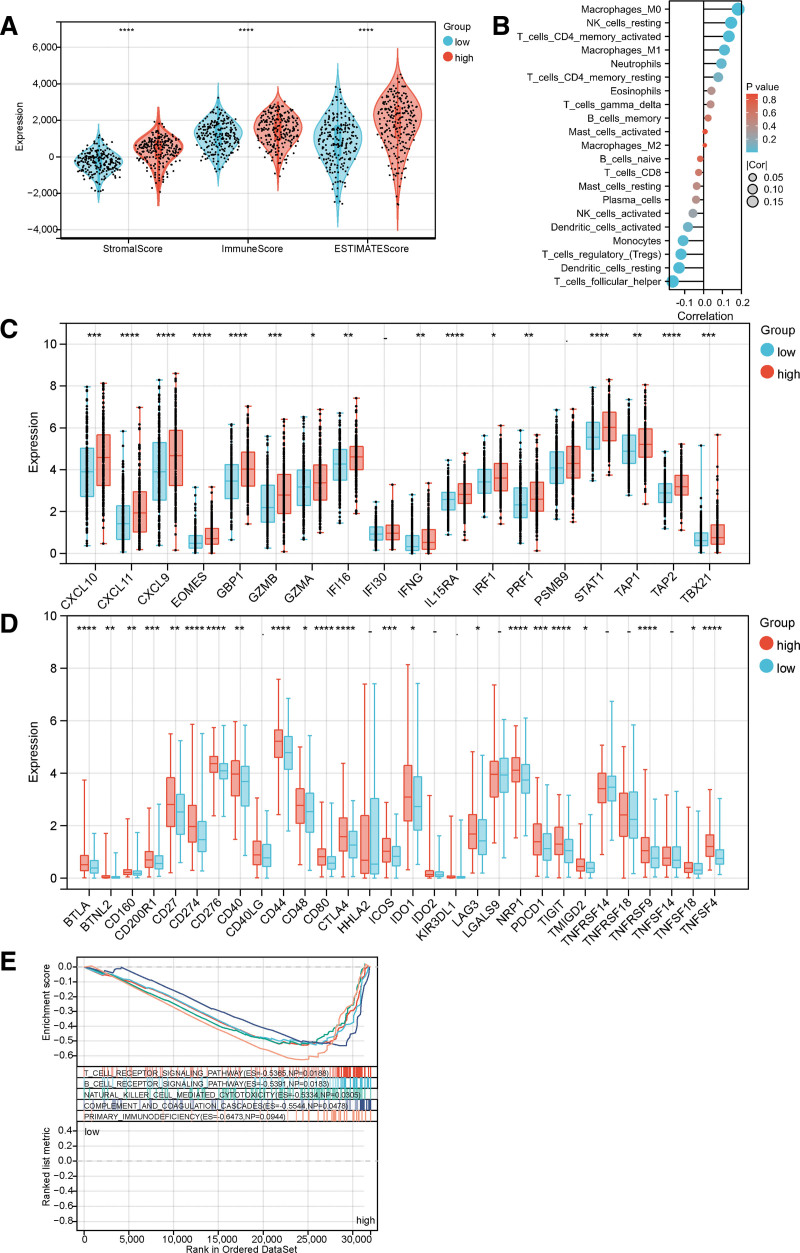
The relationship of LAMB1 expression with the immune microenvironment. (A) The comparison of stromal, immune, and ESTIMATE scores between LAMB1 high and low subtypes. (B) The correlation between LAMB1 expression and relative abundance of immune cell types. (C) T cell effectors and (D) The immune checkpoint genes differentiate between the LAMB1 high and low expression groups. “*”: *P* < .05. “**”: *P* < .01. “***”: *P* < .001. “*****”: *P* < .0001. “–”: not significant. (E) GSEA analysis identifies the underlying signaling pathway related to LAMB1.

Next, the relationship between LAMB1 expression and the immune microenvironment was analyzed. As shown in Figure 7A, the immune score was higher in the LAMB1 high-expression group than in the low-expression group. Through difference and correlation analysis, we observed that LAMB1 expression was positively associated with the immune infiltration of M0 and M1 macrophages, activated CD4 T memory cells, resting NK cells, and resting dendritic cells. Furthermore, the expression was negatively correlated with immune infiltration of vesicular helper T cells, regulatory T cells, and monocytes (Fig. 7B). Compared with the LAMB1 low-expression group, the expression of most T cell effectors and immune checkpoints was up-regulated in the LAMB1 high-expression group (Fig. 7C and D). GSEA helped decipher the biological pathways of LAMB1 gene expression. Differential expression of the LAMB1 gene is involved in immune-related pathways, such as T cell and B cell receptor signaling, natural killer cell-mediated cytotoxicity, complement and coagulation cascades, and primary immunodeficiency signaling pathways (Fig. 7E).

## 4. Discussion

LUAD is a malignant tumor with high morbidity and mortality. Although the combined application of surgery, chemotherapy, and targeted therapy has some effect on patients, the 5-year survival rate of LUAD patients is very low. Therefore, a new individualized treatment approach is urgently required to prolong the patient’s survival time. In recent years, immunotherapy, particularly immune checkpoint blockade therapy, has been considered a significant breakthrough in treating LUAD and is one of the most promising cancer treatments.^[11]^ Our study used a variety of bioinformatics methods. First, 3 LUAD-related microarray datasets were retrieved from the GEO database to obtain DEGs. These DEGs were enriched in the cell cycle, cell ECM receptor interaction, protein digestion, and absorption, p53 signaling pathway, complement and coagulation cascade, tyrosine metabolism, etc. The ECM is a structural TME component beneath the epithelial layer surrounding connective tissue cells.^[12]^ ECM can account for nearly half of tumor masses in solid tumors and is correlated with poor patient survival.^[13]^ The ECM receptor interaction signaling pathway is vital in tumor occurrence and development.^[9]^ Studies have indicated that ECM is up-regulated in prostate cancer tissues^[14]^ and is associated with tumor invasion and metastasis.^[15]^ ECM can enhance the development of epithelial-mesenchymal transition in intestinal cancer cells.^[16]^

Our study identified 2 ECM subgroups based on consensus clustering of genes involved in ECM receptor interaction pathways. The results indicated that low ECM subtypes were related to favorable clinical outcomes for patients, while high ECM subtypes were associated with higher immune scores. Immune cell infiltration analysis revealed that the infiltration degree in various immune cells was significantly correlated with ECM-related gene expressions. In addition, we constructed and validated a prognostic risk signature of 14 selected ECM-related genes, classifying the LUAD patients into high-risk and low-risk groups. These risk features can significantly predict the OS of patients and may serve as independent prognostic indicators in LUAD patients. By displaying the Venn diagram, we identified 2 risk-related genes LAMB1 and ITGB1. ITGB1, whose full name is integrin subunit beta 1, has been reported as the prognostic value in NSCLC,^[17]^ and it was shown to be correlated with lymph node metastasis.^[18]^ ITGB1 inhibition has been shown to decrease lung cancer invasion and metastasis in vitro and in vivo.^[19]^ In our work, we observed that the LAMB1 gene was significantly correlated with the survival rate of LUAD patients in the 3 datasets by performing LASSO regression analyses on the GSE68465 and GSE31210 datasets.

The LAMB1 protein is highly expressed in exosomes secreted by cells with high prostate cancer transfer ability using proteomic sequencing.^[20]^ Studies have observed that LAMB1 enhances the progression, invasion, and migration of gastric cancer cells through the ERK/c-Jun axis^[21]^ and can elevate the invasion of liver cancer cells through the PDGF/La axis.^[22]^ In addition, high expression of LAMB1 can advance glioma growth and metastasis of oral squamous cell carcinoma.^[23,24]^ We observed that the expression of LAMB1 in LUAD samples was significantly lower than in normal lung tissue based on the TCGA database analysis. Moreover, the LUAD patients with lower LAMB1 expression had shorter survival times. GSEA analysis indicated that LAMB1 was associated with various immune-related pathways. Thus, LAMB1 could be a potential prognostic molecule in non-small cell lung cancer. Meanwhile, the LAMB1 expression level was correlated with the expression of tumor immune infiltration, immune checkpoints, and T cell effectors, indicating the immunomodulatory biomarker potential of LAMB1.

Although the model has been validated in many ways, some limitations must be addressed. First, the data used is obtained from TCGA and GEO databases. Therefore, additional validation of the applicability of this model in larger patient populations is required. Second, cancer databases and bioinformatics have demonstrated a link between LAMB1 and LUAD-infiltrating immune cells. Thus, more blood samples are necessary for RT-PCR and immunohistochemical analysis to determine the function and mechanism of LAMB1 based on multiple perspectives. Third, we only focused on LAMB1 expression. However, it cannot be inferred that LAMB1 affects the prognosis of LUAD directly or indirectly using other driver genes. Finally, multicentric studies are required to validate the tumor-promoting or suppressing effects of the LAMB1 gene.

## 5. Conclusion

Based on bioinformatics analyses, our study discovered the relationship between the ECM receptor interaction pathway and the immune microenvironment in LUAD. In addition, the LAMB1 gene was screened out, which could become a new prognostic biomarker by altering the TME in LUAD and is expected to be a potential immunotherapy target. However, the study has certain limitations. Despite rigorous bioinformatics analysis, no experimental confirmation could be performed on the impact of ECM receptor interaction pathways on the immune microenvironment. Therefore, in vivo and in vitro studies are required to determine the specific mechanism of LUAD immunometabolic regulation, which will be the focus of our future research.

## Author contributions

**Conceptualization:** Ankang Hu.

**Data curation:** Ankang Hu.

**Formal analysis:** Tingjun Liu.

**Funding acquisition:** Tingjun Liu, Jing Liu, Quangang Chen.

**Investigation:** Quangang Chen, Lianlian Wu.

**Methodology:** Lianlian Wu, Lingzhi Zhang.

**Project administration:** Lingzhi Zhang, Dandan Qiao.

**Resources:** Dandan Qiao.

**Software:** Zhutao Huang.

**Supervision:** Zhutao Huang.

**Validation:** Tianyuan Lu.

**Visualization:** Tianyuan Lu.

**Writing – original draft:** Jie Wang.

**Writing – review & editing:** Jie Wang.

## Supplementary Material

**Figure:**